# EpicTope: narrating protein sequence features to identify non-disruptive epitope tagging sites

**DOI:** 10.1101/2024.03.03.583232

**Published:** 2024-03-11

**Authors:** Joseph Zinski, Henri Chung, Parnal Joshi, Finn Warrick, Brian D. Berg, Greg Glova, Maura McGrail, Darius Balciunas, Iddo Friedberg, Mary Mullins

**Affiliations:** 1 Department of Cell and Development Biology, University of Pennsylvania Perelman School of Medicine; 2 Department of Veterinary Microbiology and Preventive Medicine, Iowa State University; 3 Program in Bioinformatics and Computational Biology, Iowa State University; 4 Department of Biology, Temple University; 5 Department of Genetics, Development and Cell Biology, Iowa State University; 6 Institute of Biotechnology, Life Sciences Center, Vilnius University

## Abstract

Epitope tagging is an invaluable technique enabling the identification, tracking, and purification of proteins in vivo. We developed a tool, EpicTope, to facilitate this method by identifying amino acid positions suitable for epitope insertion. Our method uses a scoring function that considers multiple protein sequence and structural features to determine locations least disruptive to the protein’s function. We validated our approach on the zebrafish Smad5 protein, showing that multiple predicted internally tagged Smad5 proteins rescue zebrafish *smad5* mutant embryos, while the N- and C-terminal tagged variants do not, also as predicted. We further show that the internally tagged Smad5 proteins are accessible to antibodies in wholemount zebrafish embryo immunohistochemistry and by western blot. Our work demonstrates that EpicTope is an accessible and effective tool for designing epitope tag insertion sites. EpicTope is available under a GPL-3 license from: https://github.com/FriedbergLab/Epictope

## Introduction

Scientists rely on antibodies with high sensitivity to specifically recognize proteins for a multitude of functions, including visualizing subcellular localization, assessing expression levels, mapping cellular trafficking, and identifying physical interactions. Unfortunately, high quality antibodies to the majority of the vertebrate proteome are not commercially available. Some companies have produced antibodies to proteins but the vast majority do not recognize the protein in *situ* or lack specificity. Similarly, endeavors by labs to generate custom antibodies is a time- and resource-consuming process that often does not succeed, leading to a huge loss of research resources. A more reliable option is to engineer an epitope-tagged version of the protein; however, even this approach has drawbacks. Simple N- and C-terminal tags can often fail when the ends are functionally or structurally important (Zordan et al., 2015). Conversely, generating libraries of internal tags via transposon mutagenesis is effective but laborious (Zordan et al., 2015). There is a pressing need for accessible, accurate, validated algorithms to predict sites for epitope tagging that will not disrupt protein function.

There are a bevy of available online resources that could potentially be leveraged to help predict suitable epitope-tag locations in proteins of interest including sequence conservation (MUSCLE), predicted secondary structure (AlphaFold2), predicted solvent accessibility, and predicted disordered regions (IUPred). A few studies by our lab and others have shown promising predictive results by using sequence conservation (Burg et al., 2016, Gibb et al., 2018) and a combination of sequence conservation and surface accessibility (Oesterle et al., 2017). These and other genetic features have been successfully used to build predictive models for disease causing frameshift mutations in humans (Folkman et al., 2015; Pagel et al., 2019).

Here, we provide a computational tool, EpicTope, which integrates predictions of tertiary structure, secondary structure, solvent accessibility, disordered binding regions, and evolutionary conservation to predict optimal sites for epitope tagging without disrupting function. To test its efficacy, we use EpicTope to predict the best sites to epitope tag the Smad5 protein in zebrafish. Smad5 is an essential downstream transcription factor of the TGF-β//BMP signaling pathway, first required developmentally to specify ventral-lateral axial cell fates in zebrafish. It is highly conserved evolutionarily: amino acid sequences of zebrafish, chicken, and human Smad5 are 90% identical (Hata & Chen, 2016), indicating high conservation pressure and underscoring how difficult it could be to engineer a functional epitope-tagged protein. We constructed zebrafish Smad5 variants with V5-tags at two internal sites predicted to be functional by EpicTope and Smad5 variants with N- and C-terminal V5-tags with lower predicted functionality for comparison. We show that internally tagged Smad5 can rescue zebrafish *smad5* mutant embryos, while the N- and C-terminal tagged Smad5 cannot. We then show that both of the internally-tagged Smad5 proteins are nuclearly localized in the presumptive ventral region of the zebrafish gastrula. Together, our work provides an accessible tool for predicting optimal epitope-tag sites and validates its predictions by generating two functional epitope-tagged zebrafish Smad5 proteins.

## Results and Discussion:

### EpicTope prediction for Smad5

**Fig 1.**
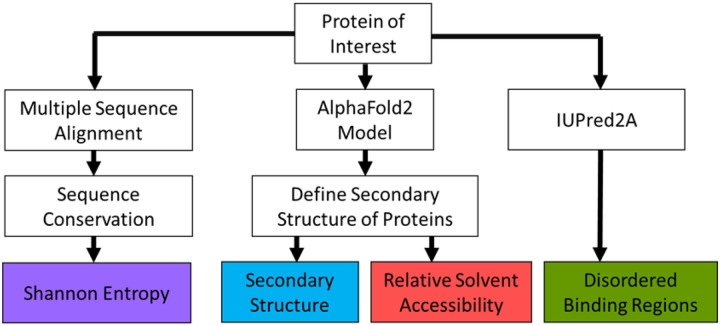
Scoring Scheme. To calculate our scoring function, we first identify a protein of interest by its corresponding Uniprot entry. We then retrieve the amino acid sequence (AA) and AlphaFold2 predicted PDB structure. The PDB structure is then used in the DSSP algorithm to determine its secondary structures and relative soluble surface area along its sequence. We use a multiple sequence alignment of the AA sequence with its homologs in seven other species to determine the sequence conservation. The UniprotID is used to retrieve the ANCHOR2 score, a measure of disordered protein binding regions, from the IUPRED2A web server.

**Fig 2.**
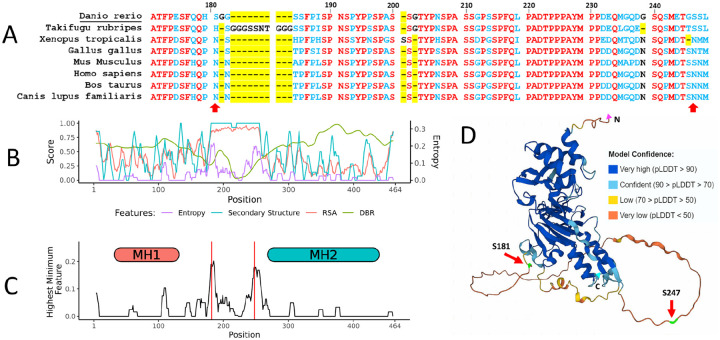
Smad5 EpicTope Features and Prediction. We calculated EpicTope predictions for Smad5, UniprotID: Q9W7E7. A) Multiple sequence alignment for Smad5 positions 170–250. Amino acids identical between all species are shown in red, and differences in one or more are in blue. Absent amino acids (length variation) are highlighted in yellow. Arrows indicate position of tag inserts, position indices are labeled in reference to *Danio rerio*. B) Shannon entropy, secondary structure, relative solvent accessibility (RSA) and disordered binding region (DBR) features used in EpicTope prediction. Features are normalized to a 0–1 scale. A higher feature score indicates suitability for tagging. C). We plot the minimum or lowest valued features at each position, and subsequently select the position with the highest minimum values (181 and 247) as predicted tag insertion sites. Feature values were averaged along a sliding window of 7 amino acids. Positions 181 and 247 are highlighted with red vertical lines. D) Smad5 AlphaFold2 predicted structure; red arrows indicate EpicTope predictions for tagging positions, highlighted in green. Disordered regions are characterized by a lower pLDDT value, a measure of model confidence. C and N termini are labeled with blue and pink triangles.

We established a computational tool, EpicTope, to predict non-disruptive epitope tagging sites within vertebrate protein sequences (Fig 1). EpicTope integrates predicted or known secondary structure, relative solvent accessibility, entropy, and IUPred2A predictions of disordered binding regions for a protein of interest, which yields a relative score for an epitope insertion at each amino acid along the protein sequence. We apply EpicTope to zebrafish Smad5 (Fig 2).

The Smad5 amino acid sequence is highly conserved across model organisms (Fig S1), with less conserved regions varying in one or more amino acids between positions 179–193 and 243–250 (Fig 2A). AlphaFold2 predicts a disordered structure from 166–258 at high confidence, with structured regions flanking this area (Fig 2B). Using the multiple sequence alignment, AlphaFold2 structure, and predictions from IUPred2A, we calculated Shannon entropy, secondary structure, relative solvent accessibility (RSA), and disordered binding region (DBR) feature scores for each position (Fig 2C). While the 166–258 region scores highly in both secondary structure and relative solvent accessibility scores, there is a predicted disordered binding region (and therefore decreased suitability for tagging) in a narrow region between 221–235 (Fig 2B, green line). We predict tagging suitability by taking the minimum score at each position of our selected features, and then identifying the highest scoring positions along the sequence length. With this approach, we identified the S181 and S247 positions as most suited for tag insertion (Fig 2C,D).

We then sought to experimentally validate the efficacy of EpicTope’s predicted tagging sites for Smad5. To test both whether tagging Smad5 at the predicted sites preserves protein function and whether the V5 is accessible to antibody binding, we created two constructs that inserted V5 tags into Smad5 at the top two predicted sites (S181 and S247). To compare to those, we also created constructs with V5 tags inserted at the N- and C-terminal ends of the protein, regions that are better conserved and more ordered than S181 and S247 (Fig. 2). S181 and S247 are at the start and end of the linker region of Smad5, a disordered region (Fig. 2D), which has CDK8/9 phosphorylation sites for MAP kinases (Kretzschmar et al. 1999). The linker region connects the well-conserved MH1 and MH2 domains (Fig. 2C). The MH1 domain is essential for nuclear localization and DNA binding, while the MH2 domain mediates a slew of protein interactions such as receptor association and Smad-Smad binding (Derynck and Budi 2019, Macias et al 2015). Disrupting any of these core functions interferes with the ability of Smad5 to transduce BMP signaling (Macias et al 2015).

### EpicTope-Predicted Smad5 Tags Preserve Protein Functionality

**Figure 3:**
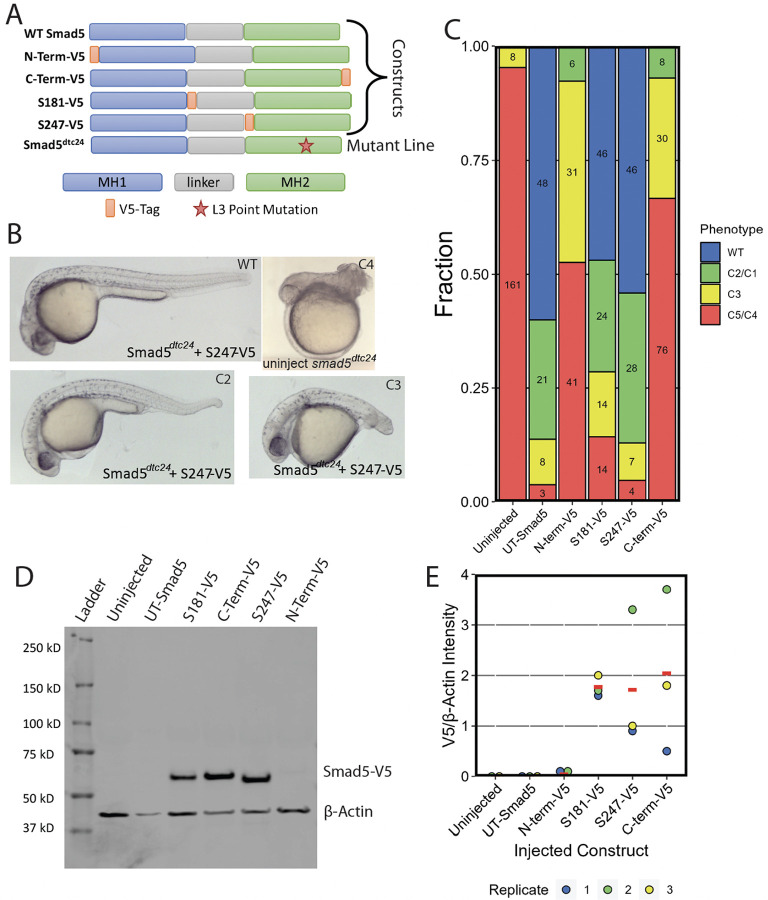
Internally V5 Tagged Smad5 Rescues Smad5 Mutant Embryos. A) Domain map of construct and mutant Smad5 proteins used. B) Representative images of 30 hpf embryos from a cross between *smad5*^*dtc24*^/+ heterozygotes injected with 150 pg of *smad5-S247-V5* RNA. C) Quantification of *smad5*^*dtc24*^+/− heterozygous in-cross injected with 150 pg of untagged or V5-tagged *smad5* RNA. The dorsalized classes C1-C5 shown in panel B are the standardized scoring scale used from Mullins et al, 1996. D) A western blot using anti-V5 and anti-beta-Actin antibodies of embryos injected with V5-tagged or untagged constructs. Seven 6 hpf (shield stage) embryos were used in each lane. E) Quantification of western blots from 3 biological replicates of injections, Replicate 1 (RNA batch 1) and Replicates 2,3 (RNA batch 2) were from different RNA synthesis reactions. The red dash is the mean.

To test whether the S181-V5 and S247-V5 Smad5 proteins are still functional and able to transduce BMP signaling, we performed a rescue experiment on zebrafish embryos deficient for Smad5. BMP signaling functions as a morphogen patterning the cells along the dorsal-ventral embryonic axis of all vertebrates in a concentration-dependent manner during the late blastula and gastrula stages of development (Madamanchi et al., 2021; Zinski et al., 2017). Zebrafish dorsal-ventral axial patterning by BMP signaling has been extensively studied. Embryos with progressively reduced BMP signaling show progressively greater degrees of dorsalization due to the loss of ventral tissue fates and expansion of dorsal ones (Mullins et al., 1996; Mintzer et al., 2000; Tuazon et al., 2020; Greenfeld et al., 2021). The early onset and progressive nature of this BMP-dependent process makes it ideal for performing quantitative rescue experiments. Here we use the *smad5*^*dtc24*^ allele that carries a dominant maternal antimorphic (dominant negative) *smad5* allele in the L3 loop of the Smad5 MH2 domain that mediates Smad-Smad interaction (Hild et al., 1999). *smad5*^*dtc24*^ heterozygous mothers produce strongly dorsalized embryos that are severely deficient in BMP signaling activity (Kramer et al., 2002; Mullins et al., 1996) (Fig. 3B). Though the *smad5*^*dtc24*^ allele is antimorphic, it can be rescued by WT Smad1/5 (Hild et al., 1999; Nguyen et al., 1998).

To test the functionality of our Smad5 constructs, we injected embryos from *smad5*^*dtc24*^ heterozygous females with mRNA made from the S181-V5, S247-V5, N-terminal, C-terminal, or untagged (UT)-*smad5* constructs. We assessed the degree of phenotypic rescue at 30 hpf (Fig. 3, S3). We evaluated the level of dorsalization using the scoring scale from Mullins et al., 1996. Both the N- and C-terminally tagged Smad5 showed minimal rescue, consistent with these tag locations disrupting Smad5 functionality (Fig. 3B,C). Conversely, both S181-V5 and S247-V5 Smad5 rescued embryos to phenotypic ratios comparable to untagged Smad5 (Fig. 3B,C). This shows that the internally-V5-tagged Smad5 proteins are similarly functional to WT Smad5 protein, while the N- and C-terminally tagged Smad5 are not.

To test whether the V5 tag interferes with protein stability, we performed western blot analysis on early gastrula embryos expressing untagged or the V5-tagged Smad5 proteins. We found that the V5-tagged Smad5 protein was present at similar levels in the S181-V5, S247-V5, and C-terminal-V5 Smad5 expressing embryos, whereas only a faint band was evident in the N-terminal-V5 Smad5 expressing embryos (Fig. 3D, Fig. S2). When quantified relative to the B-Actin control, all V5-tagged Smad5 proteins were consistently present, while no V5 tagged protein was detected in the uninjected and untagged Smad5 control conditions (Fig. 3D), as expected. This shows that S181-V5, S247-V5, and C-terminally V5-tagged Smad5 proteins are stable and present in injected embryos from *smad5*^*dct24*^ females. Meanwhile N-terminally V5-tagged Smad5 is much less abundant, indicating its stability may be affected by the presence of the tag at the N-terminus.

### EpicTope-Predicted Smad5 Tags are Accessible in Embryos to Antibody by Immunofluorescence

**Figure 4:**
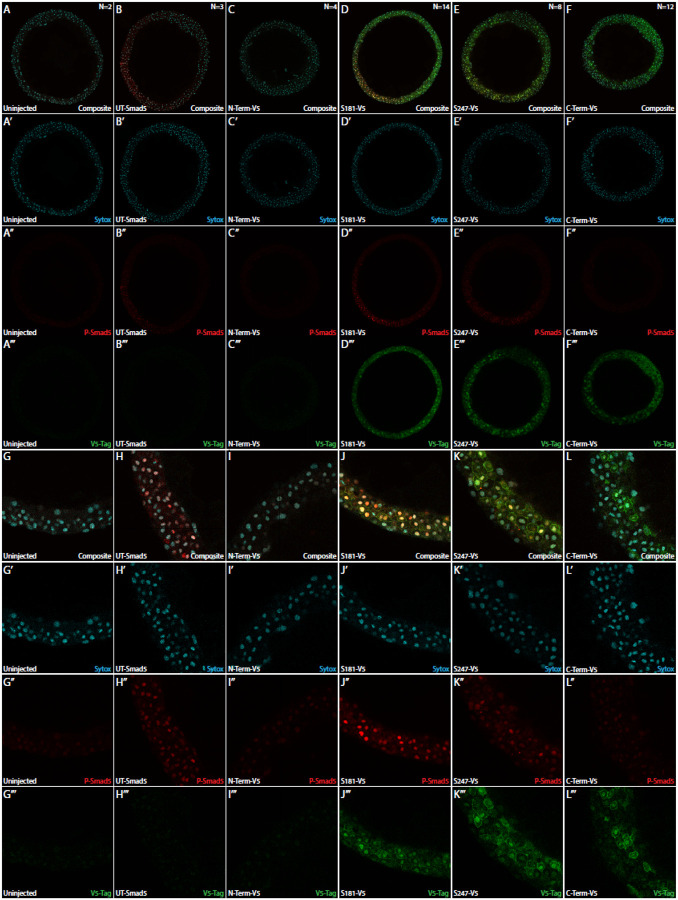
Internally Tagged Smad5 Colocalizes With Nuclear Phospho-Smad5. Smad5^dtc24^ embryos were injected with 150 pg of V5-tagged or untagged Smad5 and fixed at 5.7 hpf (germ ring stage). Embryos were immuno-stained for V5 (green), P-Smad5 (red), and for Sytox Green (DNA, blue). (A-F) Representative 560 um × 560 um × 2.2 um confocal slices near the margin of the embryo. (G-L) Zoomed in 140 um × 140 um × 2.2 um sections of the embryos shown in A-F.

We then tested whether a single V5 tag inserted at S181 or S247 is sufficient for detection of subcellular localization of Smad5 by immunofluorescence. As in Fig 3, we injected embryos from *smad5*^*dtc24*^ heterozygous females with S181-V5, S247-V5 or UT-Smad5. We immunostained embryos for the V5-tag, Phospho-Smad5 (P-Smad5), and Sytox Green (a DNA marker) and acquired confocal z-stacks at 25x zoom, a protocol we previously used to quantify changes in BMP pathway mutants (Zinski et al., 2017, 2019). Consistent with the rescue results shown in Figure 3C, we observed a WT-like gradient of P-Smad5 in untagged, S181-V5, and S247-V5 Smad5 expressing embryos but not in uninjected, C-terminal, or N-terminal tagged Smad5 embryos (Fig 4A”–F”). Consistent with the western blot analysis shown in Figure 3D, we observed V5-tagged protein in the S181, S247, and C-terminal V5-tagged Smad5 injected embryos but not in uninjected or N-terminal V5-tagged Smad5 injected embryos (Fig. 4A’”–F’”).

Receptor Smad proteins such as Smad5 reside in the cytoplasm until they are phosphorylated by a BMP Type I receptor (Hill, 2009). Once phosphorylated, receptor Smads rapidly accumulate within the nucleus, activating BMP target gene expression (Hill, 2009; Schmierer & Hill, 2005). We sought to determine if V5-tagged Smad5 also localizes to the nucleus. We observed nuclei in the ventral region of injected embryos where extracellular BMP ligand concentrations should be high. Strong nuclear P-Smad5 was present in ventral nuclei of UT, S181-V5, and S247-V5 injected embryos, but only faintly present in nuclei of uninjected, N-term-V5, or C-term-V5 injected embryos (Fig. 4G”–L”). V5-tagged Smad5 showed membrane, cytoplasmic, and nuclear localization in S181-V5, S247-V5, and C-term-V5 injected embryos (Fig. 4J’”–L’”). These results show that the internally-tagged V5 domain is accessible for immunofluorescence microscopy.

Together, our data show that EpicTope can predict locations in a protein to insert epitope-tags that do not interfere with protein function and are accessible for immuno-histochemistry and - fluorescence microscopy. We used the predictions to generate internally-tagged Smad5 proteins that can be used for subcellular Smad5 localization studies and potentially to perform CHIP-seq to determine BMP direct targets. Introducing ALFA-tags to these predicted sites could enable applications ranging from single-molecule live imaging to cell labeling for FACS sorting (Götzke et al., 2019; Igreja et al., 2022; Westlund et al., 2023).

## Methods:

### Scoring Function

We used a scoring function based on four key protein features; sequence conservation, secondary structure, solvent accessibility, and disordered binding regions (Fig 1) to determine the ideal epitope tagging sites. A query protein is first identified by its UniprotID, and the sequence and AlphaFold2 predicted structure are retrieved through Uniprot’s API (Coudert et al., 2023). We determine the query’s sequence conservation by measuring the Shannon entropy (Eq. 1) at each position in a multiple sequence alignment (MSA) with homologous proteins (Shenkin et al., 1991).


(Eq. 1) $$H=-\overset{20}{\underset{a}{\sum }}❑p\left(x_{a}\right){\text{log}}_{2}p\left(x_{a}\right)$$

We identify homologs with BLAST, using the best hit match by lowest E value against a diverse set of vertebrate organisms; *Bos taurus, Canis lupus, Gallus gallus, Homo sapiens, Mus musculus, Takifugu rubripes, and Xenopus tropicalis* (Altschul et al., 1990), and then aligning these sequences with Muscle (Edgar, 2004). The Shannon Entropy (*H*) at each position is a function of the probability for a given amino acid (*a*) to appear at that position (*X)* in the MSA, summed over all possible amino acids. We calculate secondary structures for the query protein with Define Secondary Structure of Proteins (DSSP), a tool for annotating secondary structure elements from protein structures (Kabsch & Sander, 1983). DSSP additionally provides an estimate of the solvent accessible surface area (*SASA*) for each position, and we calculate the relative solvent accessibility (*RSA*) by normalizing this estimate by the maximum solvent accessibility (SA_max_) for each amino acid (Eq. 2,(Tien et al., 2013)).


(Eq. 2) $$RSA=\text{SASA}/SA_{max}$$

We retrieve estimates of the query protein’s predicted disordered protein-protein interaction regions from the IUPred2A web server, which uses ANCHOR2 for its prediction (Mészáros et al., 2018). After calculating and retrieving the key features, we normalize the values to a 0–1 scale. We then divide the Shannon entropy by 4.32, the maximum possible entropy, in bits, over 20 amino acids (log_2_(20) = 4.32). We bin the predicted secondary structure at each position (*X*_*s*_) into a numeric value (*SS*) based on expected sensitivity to tagging (Eq. 3).


(Eq. 3) $$SS=1\;if\;X_{s}\in \left\{C\right\},0\;if\;X_{s}\in \left\{\text{GHIE}\right\},0.5\;\text{otherwise}$$

We expect defined structures such as alpha helices and beta strands (*GHIE*) to be sensitive to disruption by inserted sequences, and assign predicted these locations a 0 score. In contrast, when no secondary structure is assigned (*C*) it suggests that tag insertion would be less disruptive to the structure. Therefore, C positions were assigned a score of 1. The ANCHOR2 disordered region binding score (*BR*) is the probability for a residue position to participate in a protein binding interaction. To avoid disrupting predicted protein interactions, we use 1 - BR as input in our scoring function. After normalization, a sum score (*S*) is calculated as the summation of all features, with optional weights (*w*) for each variable, for each position *i* (Eq. 4).


(Eq. 4) $$S_{i}=w_{1}\frac{H_{i}}{4.32}+w_{2}SS_{i}+w_{3}RSA_{i}+w_{4}(1-BR_{i}{)}_{❑}$$

As an initial proof of concept, we set the Shannon entropy weight to 1.5 and all other weights to 1. These weights can be tuned to find an optimal contribution of each feature for ideal internal epitope tag insertion site prediction. For each position, we calculate a tagging score for any position *i* (*E*_*i*_), or the minimum value between all features (Eq. 5). We then seek out positions along the sequence where this minimum score is the highest. We use this approach to identify regions where all chosen features indicate tagging suitability.


(Eq. 5) $$E_{i}=min\left(\frac{H_{i}}{4.32},SS_{i},RSA_{i},\left(1-BR_{i}\right)\right)$$

### Rescue with WT and V5-Tagged Constructs

The zebrafish used for the rescue experiment were *smad5*^*dct24*^ heterozygotes, ID ZDB-ALT-980203–795. Embryos from *smad5*^*dct24*^ heterozygous females produce embryos with C4 or C5 dorsalized phenotypes according to the scoring scale from Mullins et al. 1996. Embryos from this cross were injected at the 1-cell stage with 150 pg of RNA. RNA was made using the N-terminal-V5, S181-V5, S247-V5, or C-terminal-V5 plasmids generated for this papers or from a pCS2+ plasmid encoding Untagged-Smad5. Rescue was assessed at 30 hours-post-fertilization using the scoring scale from Mullins et al. 1996.

### Microscopy and Image Analysis

Embryos from a *smad5*^*dct24*^ heterozygote incross were injected a the 1-cell stage with 150 pg of RNA from N-terminal-V5, S181-V5, S247-V5, or C-terminal-V5, and UT-Smad5. Embryos were fixed at 6 hours post fertilization in 4% PFA PBS. We then performed immunohistochemistry for the V5-Tag and P-Smad5 to quantify nuclearly localized P-Smad5 and V5-Tag as previously described (Zinski et al., 2019). The P-Smad5 antibody was obtained from Cell Signaling Technology #13820. The V5-tag antibody was obtained from Invitrogen R960–25. Imaging was performed using an Zeiss LSM 880 confocal microscope with a LD LCI Plan-Apochromat 25×/0.8 Immersion Corr DIC M27 multi-immersion lens and a Plan-Apochromat 63x/1.40 Oil DIC M27 lens.

### Western Blot

Embryos were dechorionated and the yolks manually removed at 6 hours post fertilization. Samples were flash-frozen in liquid nitrogen and stored overnight at −80C. Seven embryos were pooled for each condition. Samples were denatured at 95C for 5 minutes in 2X Laemlli buffer (65.8 mM Tris-HCl, pH 6.8, 2.1% SDS, 26.3% (w/v) glycerol, 0.01% bromophenol blue) and 5% beta-mercaptoethanol. Denatured protein in Laemlli buffer was loaded into a 4–15% gradient SDS-Page gel in a Mini Trans-blot cell (BioRAD) with running buffer (in dH2O, 25 mM Tris, 192 mM glycine, 0.1% SDS) and run at 50V for 10 minutes, followed by 100V for 80 minutes. Proteins were transferred to LF-PVDF membrane in transfer buffer (in dH2O, 20% methanol, 25 mM Tris, 192 mM glycine) at 4C at 100V for 50 minutes. The membrane was washed 3 times for 15 minutes in 1xTBST (150 mM NaCl, 10 mM Tris pH 8.0, 0.1% Tween20) then for 2 hours in 4% skim milk in TBST. The membranes were incubated in primary antibodies (1:1000 rabbit anti-β-actin (A2066 Sigma Aldrich), 1:2000, mouse anti-V5) were incubated in 4% milk in TBST O/N at 4°C, washed 3 times for 15 minutes, then incubated in secondary (1:10000 anti-mouse DyLight 680, 1:10000 anti-rabbit DyLight 800) for 40 minutes at RT in the dark. Membrane was washed 3 time in TBST and imaged on a LI-COR imaging system (LI-COR Biosciences).

## Supplementary Material

Supplement 1

## Data Availability

EpicTope is available under a GPL-3 license from: https://github.com/FriedbergLab/Epictope
