# Supplementary material for: EpicTope: narrating protein sequence features to identify non-disruptive epitope tagging sites
Source: bioRxiv. 2024 Mar 11:2024.03.03.583232. Preprint. [Version 2] doi: 10.1101/2024.03.03.583232 (PMC10979891; doi:10.1101/2024.03.03.583232)
Supplement: Supplement 1 [file media-1.pdf]

Supplementary Materials

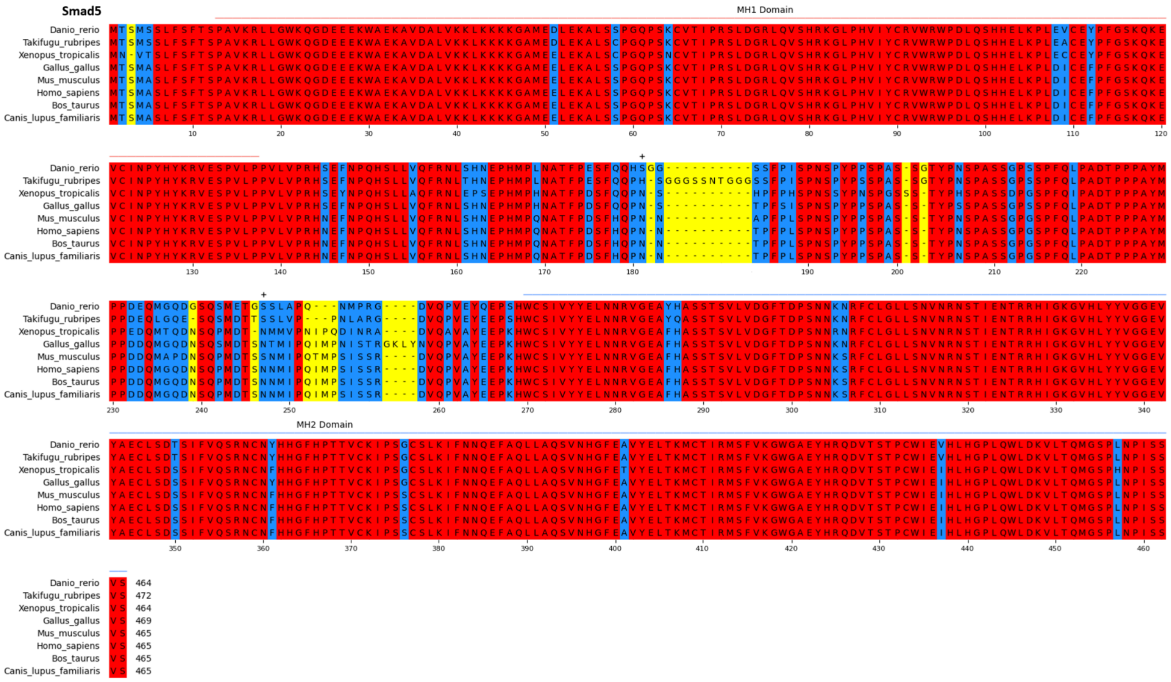

Figure S1. Multiple Sequence Alignment for *Danio rerio* Smad5. Amino acids identical between all species are highlighted in red, and differences in one or more residues are highlighted blue. Absent amino acids (length variation) are shown in yellow. Location of internally inserted *Danio rerio* Smad5 tags at S181 and 247 are labeled with a black + MH1 and MH2 domains are indicated by horizontal red and blue bars, respectively. Total length of each Smad5 protein is shown at the end of the alignment, and position indices are labeled in reference to *Danio rerio*.

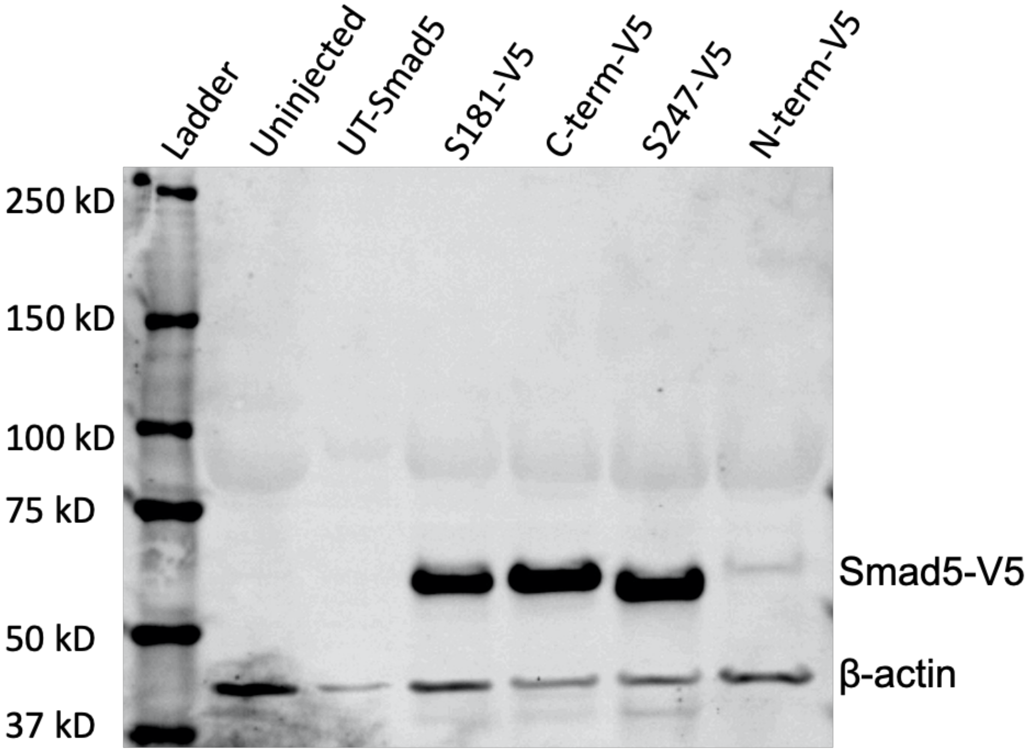

Figure S2. Longer exposure of the western blot in Figure 3 to show the N-terminal V5 tagged Smad5 band using anti-V5 and anti-beta-Actin antibodies of extracts from embryos injected with V5-tagged or untagged constructs. Seven 6 hpf (shield stage) embryos were used in each lane.

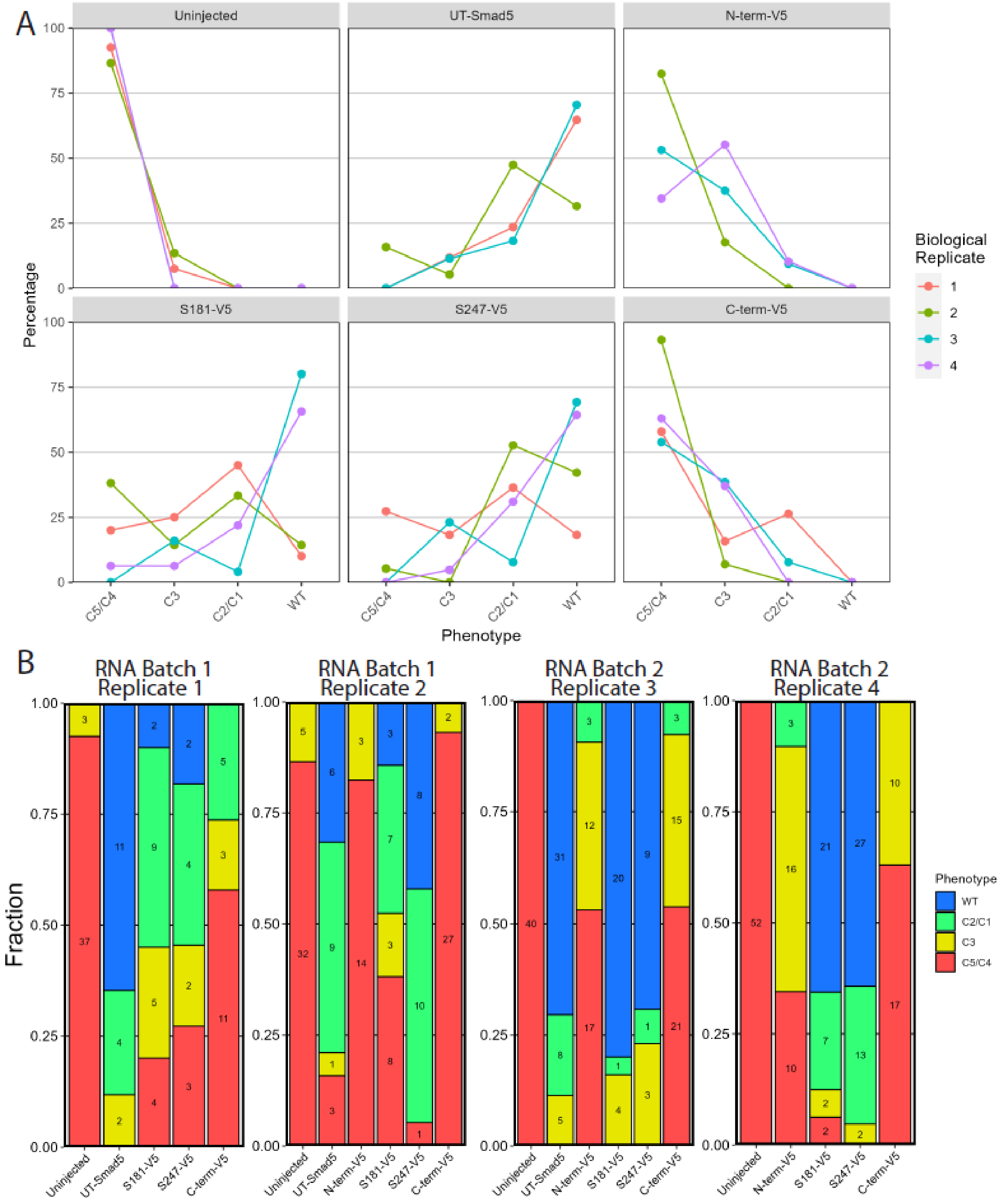

Figure S3. Biological Replicates of V5-Tagged Smad5 Rescue. Quantification of *smad5*^{dtc24}+/- heterozygous in-cross injected with 150 pg of untagged (UT) or V5-tagged *smad5* RNA. The Dorsalized classes C1-C5 shown in panel B are the scoring scale standardized in

Mullins et al, 1996. A) Points on the lines are the percentage of embryos with the phenotype specified on the x-axis. Different colored lines are different biological replicates. B) Bar graphs of the data shown in A with the number of embryos identified within each bar.
